# Prognostic value of area of calcified aortic valve by 2-dimensional echocardiography in asymptomatic severe aortic stenosis patients with preserved left ventricular ejection fraction

**DOI:** 10.1097/MD.0000000000010246

**Published:** 2018-03-23

**Authors:** Victor Chien-Chia Wu, Masaaki Takeuchi, Yasufumi Nagata, Masaki Izumo, Yoshihiro J. Akashi, Fen-Chiung Lin, Yutaka Otsuji

**Affiliations:** a Second Department of Internal Medicine, University of Occupational and Environmental Health, School of Medicine, Kitakyushu, Japan; b Division of Cardiology, Chang Gung Memorial Hospital, Linkou Medical Center, Taoyuan, Taiwan; c Department of Laboratory and Transfusion Medicine, University of Occupational and Environmental Health, School of Medicine, Kitakyushu; d Department of Cardiology, St. Marianna University School of Medicine, Kawasaki, Japan.

**Keywords:** 2D echocardiography, aortic valve calcification, severe aortic stenosis

## Abstract

We hypothesized that area of calcified aortic valve (ACAV) measured by 2D echocardiography (2DE) can predict future cardiovascular events in asymptomatic severe aortic stenosis (AS).

Multidetector computed tomography determined aortic valve calcification load is strongly associated with AS severity but has risks for radiation exposure. Quantification of ACAV by transthoracic 2DE is simple and convenient but its clinical utility has not been extensively studied.

We measured ACAV in 124 asymptomatic severe AS patients (80 ± 9 years, 45 males) with preserved left ventricular ejection fraction. ACAV was measured by planimetry from 2D zoomed long axis view of the AV at end-diastole. Patients were followed to record cardiac death (CD) and major adverse cardiovascular events (MACEs).

During a median follow-up of 232 days, 17 patients had MACE, including 8 CD. ACAV was significantly larger in patients with event compared to those without (1.14 ± 0.35 cm^2^ vs 0.87 ± 0.34 cm^2^, *P=*.0032). Using receiver operating characteristics derived ACAV of 0.79 cm^2^ as cutoff value, Kaplan–Meyer analysis showed it could discriminate high-risk group from low-risk group for future CD (*P=*.0223, *χ*^2^ = 5.22) and MACE (*P = *.0054, *χ*^2^ = 7.74).

2DE determined ACAV is straightforward and has potential to predict future cardiac events in asymptomatic severe AS patients.

## 1 Introduction

Symptomatic patients with severe calcified aortic stenosis (AS) portend grave prognosis and have class I indication for surgical or transcatheter aortic valve replacement (AVR) depending on the compelling clinical conditions.^[1,2]^ Controversy still exists regarding early surgery in asymptomatic severe calcified AS patients with preserved left ventricular ejection fraction (LVEF).^[3,4]^ Several potential variables including left ventricular mass,^[5–7]^ left ventricular mechanics,^[8–11]^ left ventricular diastolic function,^[12,13]^ and left atrial volumes^[14,15]^ have been reported to be useful parameters stratifying high risk asymptomatic severe AS patients with future cardiovascular events.

Calcific aortic valve (AV) disease is the final pathway from multiple atherosclerotic risk factors, including age, gender, hypertension, lipoprotein (a), low-density lipoprotein, and inflammation exposing on the AV.^[16–18]^ Measurement of aortic valve calcification (AVC) load with multidetector computed tomography (MDCT) has been shown to be a simple and reliable way for quantify AV weight,^[19,20]^ and efforts have been made to define specific thresholds of AVC that can discriminate true severe AS from pseudosevere AS in discordant severe AS grading.^[21]^ In addition, MDCT determined AVC load provides incremental prognostic value for survival beyond clinical and Doppler echocardiographic assessment and is considered for risk stratification in patients with AS.^[22]^ However, MDCT poses radiation risks therefore quantification of AVC is not routinely performed on regular follow-up basis. We hypothesized that calcified aortic valve area tracing on transthoracic 2D echocardiography (2DE) is another simple modality to quantify AV weight, and thus, has a potential predictor for future cardiac events. Accordingly, the aims of this study were to validate the accuracy of area of calcific aortic valve area (ACAV) measurements by 2DE against surgically explanted aortic valve weight, and determine the predictive power for future cardiac death (CD) and major adverse cardiovascular events (MACE).

## 2 Methods

### 2.1 Study subjects

2D echocardiographic data were collected in 307 patients with severe aortic stenosis who were enrolled from 2 university hospitals (University of Occupational and Environmental Health and St. Mariana University) from January 2012 to December 2013. These patients were part of our ongoing prospective study of aortic stenosis (Japanese ultrasound aortic stenosis study—prospective arm; JUST-P). After exclusion of 109 severe AS patients with symptoms and 23 patients with LVEF< 50%, and 51 patients with peak velocity <3.0 m/s, the final study population consisted of 124 asymptomatic severe AS patients with preserved LVEF. The ethics committee of each hospital approved the study protocol, and informed consents were obtained in all subjects.

### 2.2 2D transthoracic echocardiography

Routine comprehensive echocardiographic exams were performed, with special emphasis to document the severity of aortic stenosis. Zoomed parasternal long axis (PLAX) view was used for the measurement of ACAV at end-diastolic phase of cardiac cycle. Contrast levels were adjusted to best visualize and delineate the calcified areas against background of normal AV tissue or AV sclerosis. Two ultrasound machines (Philips and GE) were the platforms used to acquire echocardiographic study images in these patients. The bright area of the AV was then manually traced and area automatically measured by vendor-independent software package ProSolv (CardioVascular 4.0, Fujifilm, Indianapolis, IN).

### 2.3 Reproducibility

Intraobserver variability was assessed in the same observer 2 weeks apart by repeating the measurement of ACAV using zoomed PLAX view of AV in 15 randomly selected patients. Interobserver variability was assessed in another observer by performing these measurements in the same 15 patients. Intraobserver and interobserver variability were calculated with the absolute value of the differences between the 2 measurements in the same patient using percent of their mean.

### 2.4 Follow-up

Follow-up information in AS patients was obtained regularly in outpatient clinic. Telephone contacts to patients, physicians, and next of kin were performed, if the patient had been treated in the other hospital. The primary end-point was CD, and secondary end-point was MACE, which was defined as occurrence of CD, nonfatal myocardial infarction, and heart failure requiring admission. Patients who had received AVR were censored at the time of operation.

### 2.5 Statistical analysis

The continuous data are given by mean ± SD. The categorical data are shown as a number or percentage. In addition, the categorical variables were compared using Fisher's exact test whenever appropriate. A Student's *t*-test was carried out to test the differences in continuous variables between the 2 groups. Linear regression was used to study the relationship between 2 parameters. Univariate Cox proportional hazards model was used to identify the significant predictors of CD and MACE. Multivariate Cox proportional hazards model was used to determine the independent variables for predicting future CD and MACE. A *P*-value <.05 was considered significant. Intra- and interobserver variability values were calculated with the absolute value of the differences between the 2 measurements in the same patient using percent of their mean. Inter- and intraobserver reproducibility analyses were carried out with intraclass correlation coefficient (ICC). The statistical analyses were all performed using commercially available software (JMP, version 11.0, SAS Institute Inc., Cary, NC; SPSS, version 17, SPSS Inc., Chicago, IL).

## 3 Results

**Figure 1:**
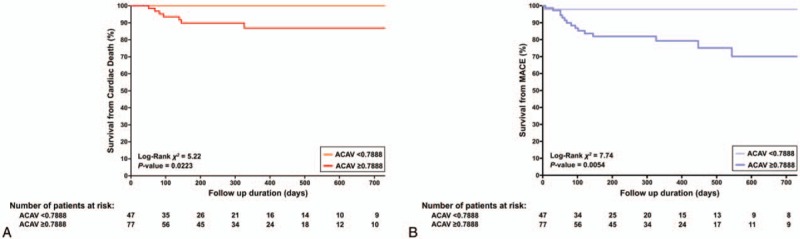
Kaplan–Meier survival analyses for cardiac death (A) and MACE (B) by ACAV cutoff of 0.7888 cm^2^. ACAV = area of calcified aortic valve, MACE = major adverse cardiovascular events.

All patients could be measured of the ACAV. Table 1 represents clinical characteristics in the study population. ACAV showed weak but significant correlation with peak velocity (*r* = 0.37, *P* <.0001), mean pressure gradient (PG) (*r* = 0.38, *P < *.0001), indexed AV area (r = 0.37, *P < *.0001). During a median follow up of 232 days (interquartile range: 91–518 days), 8 patients had CD and 17 patients had MACE. Table 2 showed echocardiographic parameters between patients with and without CD and MACE. ACAV was significantly higher in patients with cardiac events compared to those without (*P = *.0032). Receiver operating analysis revealed ACAV of 0.79 cm^2^ had a 100% sensitivity and a 41% specificity for future CD and a 94% sensitivity and a 43% specificity for future MACE. Kaplan–Meier survival analyses showed ACAV is a significant predictor for CD (*P = *.0223) and MACE (*P = *.0054) (Fig. 1). Since the number of CD and MACE were small, we sought to determine the usefulness of ACAV in univariate and 3 multivariate Cox Proportional Hazards models (Table 3). In univariate Cox models for predicting CD, history of myocardial infarction and/or coronary artery bypass graft (MI/CABG), peak velocity across aortic valve, mean pressure gradient (PG), left ventricular stroke volume index and ACAV emerged as significant, while indexed aortic valve area was not. In multivariate Cox models, ACAV still is significant for predicting CD after adjusting for MI/CABG, left ventricular stroke volume index or mean PG. In univariate Cox models for predicting MACE, peak velocity, mean PG, indexed aortic valve area (iAVA), and ACAV emerged as a significant predictor. In multivariate Cox models, ACAV still was significant for predicting MACE after adjusting for peak velocity, mean PG, iAVA.

**Table 1:**
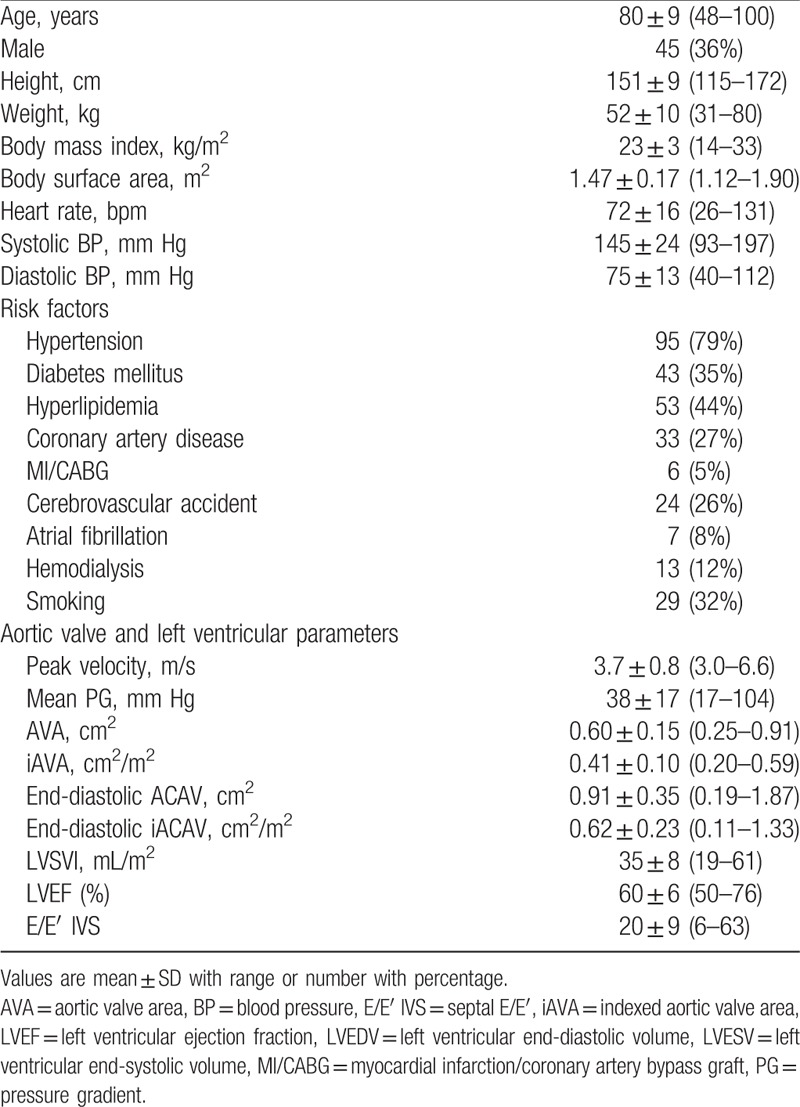
Clinical characteristics in the study subjects (n = 124).

**Table 2:**
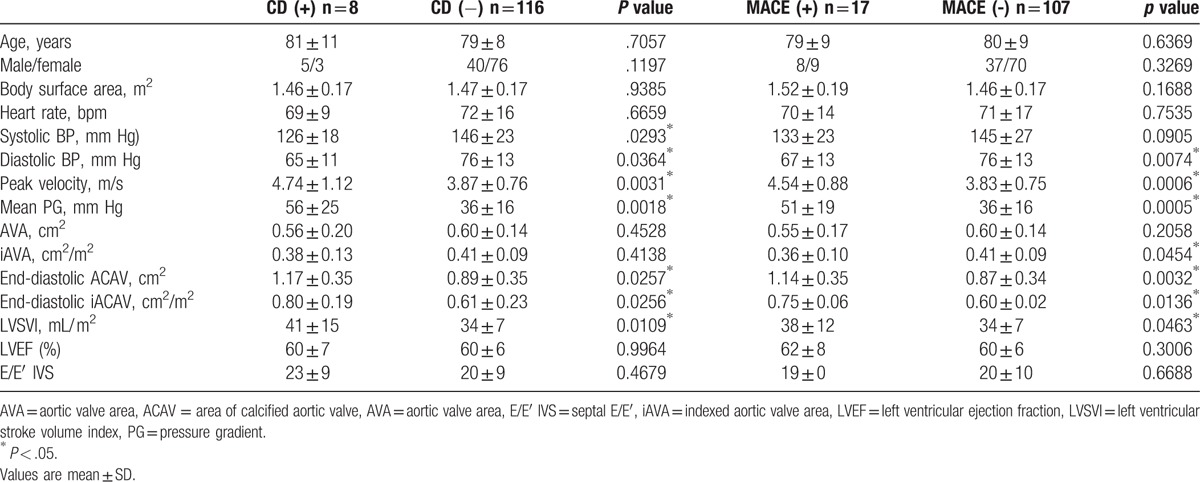
Comparison of echocardiographic parameters between patients with and without cardiac death and MACE.

**Table 3:**
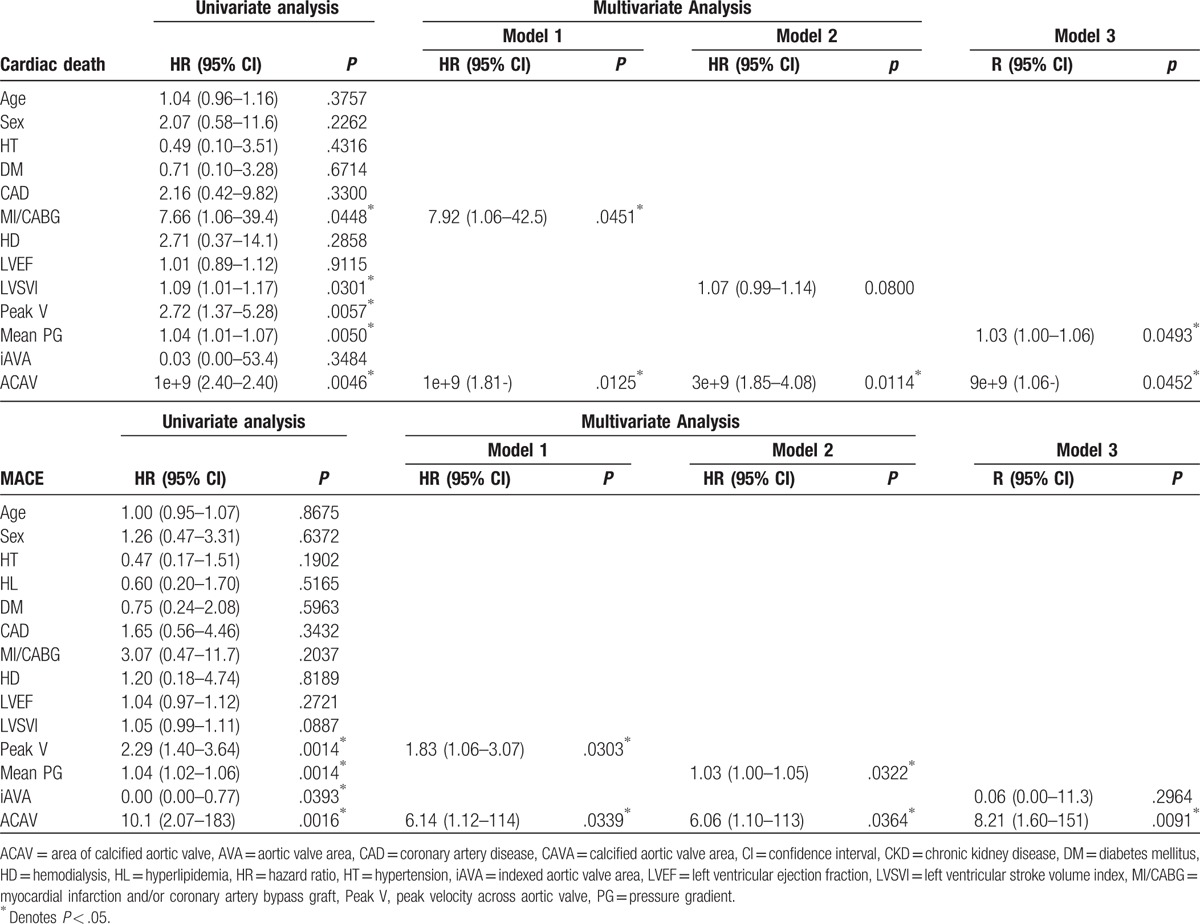
Predictors of cardiac death and MACE.

### 3.1 Observer variabilities and intraclass correlation coefficient

Intra- and interobserver variabilities of ACAV were 4.1 ± 2.7% and 9.0 ± 6.0%, respectively. Intra- and interobserver ICC were 0.97 and 0.87, respectively.

## 4 Discussion

The major findings of this study were that ROC derived cut-off value of ACAV, could discriminate high risk from low risk in asymptomatic severe AS patients for both future CD and MACE.

### 4.1 Previous study

Prior investigation of AVC mostly involved the utilization of computed tomography. In the study by Messika-Zeitoun et al,^[19]^ surgically excised AV specimens underwent tissue digestion to quantify residual calcium weight against electron-beam computed tomography (EBCT). Using EBCT to asses AVC, calcification was defined as 4 adjacent pixels with density >130 Hounsfield units at the frame of 80% of RR interval on electrocardiography. These well-defined electromechanical parameters ensured standardized AVC measurement by different operating personnel, with resultant data examine by dedicated analyze software that have benefits of minimizing human errors in the judgment of calcification area. Within the same group of researchers, Clavel et al^[21]^ used Agatston method expressed in arbitrary unit (AU) that has been well established in the quantification of coronary artery calcium level.^[23]^ In the continuation of study, although the impact of severe AVC load in patients with AS is independent predictor of overall mortality and has additive predictive value over traditional hemodynamic parameters, the enrollment included wide range of patients including severity greater or equal to mild, mean gradient ≥15 mm Hg, peak aortic jet velocity ≥2.0 m/s, or aortic valve area ≤2 cm^2^, with or without symptoms.^[22]^ However, both obesity and extensive valve calcification were still identified as potential factors that caused deterioration in both echocardiography and CT performance. While efforts have been made to define calcification size by echocardiographic means,^[24]^ the classification was at most semi-qualitative and inter-person interpretation may vary widely. These limitations make underutilization of echocardiography for the assessment of AVC.

### 4.2 Current study

Ideally, the aortic stenosis is defined AVA ≤1 and Vmax ≥4 m/s, or a mean gradient ≥40 mm Hg according to ACC/AHA and ESC guidelines.^[1,2]^ However, studies have found discrepancies frequently observed between the mean gradient and the valve area in a single patient.^[25]^ In patients with an AVA < 1 cm^2^, there are 4 flow-gradient AS categories: normal flow (NF)/low gradient (LG), NF/high gradient (HG), low flow (LF)/HG and LF/LG, whereas low flow is defined as an indexed LV stroke volume <35 mL/m^2^ and low gradient is mean trans-aortic PG <40 mm Hg.^[26]^ The new entity, “paradoxical low-flow, low-gradient” severe AS, defined as having a stroke volume index <35 mL/m^2^, mean PG <40 mm Hg, and AVA <1.0 cm^2^ or indexed AVA <0.6 cm^2^/m^2^ with LVEF ≥50%, has gained clinical interest in recent years,^[27,28]^ and in particular, the smaller body size of Japanese patients with AS may increase AVA-mPG discordance.^[29]^ As shown in Table 1, the 124 patients enrolled in this study has a mean AVA of 0.60 ± 0.15 (0.25–0.91) cm^2^, mean indexed AVA of 0.41 ± 0.10 (0.20–0.59) cm^2^/m^2^, Vmax of 3.7 ± 0.8 (3.0–6.6) m/s, and mean gradient of 38 ± 17 (17–104) mm Hg, which are frequently seen in the Japanese population and some patients may corresponded to subgroup of severe AS.

Although CT and TEE each have their advantage compared to TTE, these modalities usually are not considered first-line examination of choice due to radiation exposure by CT. TTE is in fact the standard exam for the assessment of heart size, chamber mechanics, and valvular hemodynamics and can be repeated without incurring any risks. In the MDCT study the explanted AVC score by EBCT showed strong linear correlation with valvular calcification weight.^[19]^ Since explanted calcified part of AV has higher specific gravity than explanted noncalcified part of AV, ACAV was expected to correlate better with AV weight in explanted AV with higher calcium content. In our study, we assessed the accuracy of ACAV as surrogate marker for AVC load in the explanted AV weight obtained during AVR. Therefore in this study we aimed to assess the accuracy of ACAV as surrogate marker for explanted AV weight obtained during AVR. While earlier study used only semiquantitative or qualitative evaluation on the degree of calcification of the aortic valve,^[24]^ we approached with quantitative means by tracing ACAV selected from the end-diastolic phase at onset of QRS complex from 2DE in place of more cumbersome ways of calculating AVC loading by MDCT.

Asymptomatic severe AS patients have risks for small but unpredictable sudden cardiac death that often raised significant concerns regarding early AV surgery. As shown in this study, cardiac death rates are known to worsen in patients who had more extensive ACAV. Using ROC curve derived ACAV cut-off value 0.79 cm^2^, Kaplan–Meier survival analysis showed increased risk for cardiac death with increased AVC in this highly selected group of asymptomatic severe AS patients with preserved LVEF.

In univariate analysis of Cox proportional hazards model for future CD, we found past history of MI/CABG, left ventricular stroke volume index, peak velocity, mean PG, and ACAV showed significance as independent variables. In multivariate analysis, ACAV still was significant for predicting cardiac death with separate models. In addition, in univariate analysis of Cox proportional hazard model for future MACE, we found peak velocity, mean PG, and iAVA showed significance as independent variables. In multivariate analysis, ACAV again was significant for predicting MACE. In summary, ACAV not only is a surrogate for calcified AV weight thus reflecting the pathophysiological severity of AS, but also provides useful prognostic information in asymptomatic AS patients with preserved LVEF.

Over the years, there are increasing number of variables that are available to cardiologists for risk stratification asymptomatic severe AS patients. However, overwhelming variety of parameters requires dedicated software for their quantifications. Our study has shown that 2D echocardiographic determination of ACAV is simple, feasible, and clinically relevant for future prognosis. Larger studies should be necessary to standardize the assessment by protocols and well-defined parameters.

### 4.3 Limitations

There were several limitations that should be acknowledged. First, our study has relative small study population and statistical results may be underpowered. Second, our population had a higher prevalence of small body size therefore there were a number of patients having inconsistent grading of AS according to the guidelines. However, the situation often was encountered in clinical setting in Japanese population and also was the reason to study AVC load by ACAV in risk-stratifying these intermediate severity patients. The application of our results and the generalization to other ethnicities shall require further studies. Third, the number of events was small to perform multivariate Cox regression analysis including all possible variables due to overfitting. Fourth, previous MDCT studies used cross-sectional view of AV for quantification of AVC load using all 3 AV leaflets, but our study utilized zoomed PLAX view for determination of ACAV using 2 AV leaflets. Using MDCT, best view for quantification of aortic valve calcification (AVC) load is possible since optimal cutting plane for AVC measurement can be selected retrospectively. However, in echocardiographic study the best cutting plane for AVC measurement cannot be chosen retrospectively. In two-dimensional echocardiography (2DE), obtaining clear and unobstructed images are easier in parasternal long-axis (PLAX) view with ultrasound probe oriented and fit better within the intercostal space (ICS) compared to parasternal short-axis (PSAX). In addition, in frail elderly patients, narrowed intercostal space ICS due to decreased lung capacity and tidal volume frequently prevent clear images being acquired in parasternal PSAX view. Fifth, the echo images are obtained from different ultrasound machines (Philips and GE) therefore the image quality may not be consistent, although we used vendor-independent software (ProSolv) to trace ACAV across images acquired from different platforms. Last, the mean age was 80 years and the findings may not be generalizable to younger populations. Further study is necessary for AS patients mostly consist of younger age whose PSAX view can be better assessed for the determination of area of leaflet calcification in all cusps.

## 5 Conclusion

2DE determined ACAV was a simple and reliable parameter for estimating AV weight that reflects severity of AS. ACAV was shown to be a powerful predictor for both future CD and MACE in asymptomatic severe AS patients with preserved LVEF.

### 5.1 Data access

Data are available from the University of Occupational and Environmental Health Institutional Data Access/ethics committee for researchers who meet the criteria for access to confidential data

## Acknowledgments

We would like to thank Alfred Hsing-Fen Lin for the statistical assistance during the completion of this manuscript

## 6 Author contributions

**Conceptualization:** V.C-C. Wu, M. Takeuchi.

**Data curation:** V.C-C. Wu, Y. Nagata, M. Izumo.

**Formal analysis:** V.C-C. Wu, M. Takeuchi, Y. Nagata.

**Investigation:** V.C-C. Wu, M. Takeuchi, M. Izumo.

**Methodology:** V.C-C. Wu, M. Takeuchi, Y. Nagata.

**Project administration:** M. Takeuchi.

**Software:** M. Takeuchi, Y. Otsuji.

**Supervision:** M. Takeuchi, Y.J. Akashi, F-C. Lin, Y. Otsuji.

**Writing – original draft:** V.C-C. Wu.

**Writing – review & editing:** V.C-C. Wu, M. Takeuchi.
